# Supplementary material for: Detailed molecular and epigenetic characterization of the pig IPEC-J2 and chicken SL-29 cell lines
Source: iScience. 2023 Feb 20;26(3):106252. doi: 10.1016/j.isci.2023.106252 (PMC10018572; doi:10.1016/j.isci.2023.106252)
Supplement: Data S2. Complete homer output for identified motifs in Chicken SL-29, related to Tables 5 and 6 — Homer motif analysis results for histone modifications H3K4me1, H3K4me3, H3K27ac, enhancers, and ATAC-seq of chicken SL-29 cell line. Parameters for possible false positives is as mentioned earlier for S5. [file mmc3.zip › Data_S2/S6/Chicken_SL_29/peak_files_ATAC/homerResults/motif8.similar.html]

motif8

## Information for motif8

T
C
A
G
C
A
T
G
C
T
G
A
C
T
G
A
T
C
A
G
T
C
A
G
A
G
T
C
T
A
G
C
G
A
C
T
G
T
A
C
  
Reverse Opposite:  

C
A
T
G
C
T
G
A
A
T
C
G
T
C
A
G
A
G
T
C
A
G
T
C
G
A
C
T
A
G
C
T
G
T
A
C
A
G
T
C
  

|  |  |
| --- | --- |
| p-value: | 1e-376 |
| log p-value: | -8.676e+02 |
| Information Content per bp: | 1.457 |
| Number of Target Sequences with motif | 9806.0 |
| Percentage of Target Sequences with motif | 11.27% |
| Number of Background Sequences with motif | 6359.2 |
| Percentage of Background Sequences with motif | 7.33% |
| Average Position of motif in Targets | 150.7 +/- 86.2bp |
| Average Position of motif in Background | 149.7 +/- 93.2bp |
| Strand Bias (log2 ratio + to - strand density) | -0.0 |
| Multiplicity (# of sites on avg that occur together) | 1.19 |
| Motif File: | file (matrix) reverse opposite |

### Similar de novo motifs found

|  |  |  |  |  |  |  |  |
| --- | --- | --- | --- | --- | --- | --- | --- |
| Rank | Match Score | Redundant Motif | P-value | log P-value | % of Targets | % of Background | Motif file |
| 1 | 0.748 | C G T A A C G T T C A G G T C A C A T G T C A G A G T C A G T C | 1e-296 | -682.350784 | 16.97% | 12.64% | motif file (matrix) |
| 2 | 0.622 | T A C G C T A G T A G C G T A C G T C A G T A C C G A T T G A C | 1e-211 | -487.668008 | 34.34% | 29.46% | motif file (matrix) |
| 3 | 0.611 | T C A G C G T A A G T C C T A G A G T C G T A C T G C A G A C T | 1e-125 | -288.087244 | 15.70% | 12.91% | motif file (matrix) |
